# Association between Peak Neutrophil Count, Clopidogrel Loading Dose, and Left Ventricular Systolic Function in Patients with Primary Percutaneous Coronary Intervention

**DOI:** 10.1155/2014/482763

**Published:** 2014-07-24

**Authors:** Xinyu Wang, Haiyi Yu, Zhaoping Li, Liuning Li, Youyi Zhang, Wei Gao

**Affiliations:** ^1^Department of Cardiology, Institute of Vascular Medicine, Huayuanbeilu 49, Haidian District, Peking University Third Hospital, Beijing 100083, China; ^2^Key Laboratory of Cardiovascular Molecular Biology and Regulatory Peptides, Ministry of Health and Key Laboratory of Molecular Science, Ministry of Education, and Beijing Key Laboratory of Cardiovascular Receptors Research, Beijing 100191, China

## Abstract

Inflammation plays an important role in plaque development and left ventricular remodeling during acute myocardial infarction (AMI). Clopidogrel may exhibit some anti-inflammatory properties and high loading dose of clopidogrel results in improved clinical outcomes in patients with AMI. 357 patients who received successful primary percutaneous coronary intervention from January 2008 to March 2011 in Peking University Third Hospital were included in this study. Different loading dose of clopidogrel (300 mg, 450 mg, or 600 mg) was given at the discretion of the clinician. Neutrophils reached their peak values on the first day after AMI. Higher levels of peak neutrophil and lower left ventricular ejection fraction (LVEF) were found in patients of low clopidogrel loading dose group (300 mg or 450 mg). After adjusting for the related confounders, a logistic regression model showed that low clopidogrel loading dose remained an independent predictor of low LVEF (LVEF ≤ 50%) [OR: 1.97, 95% CI: 1.03–3.79, *P* = 0.04]. Low clopidogrel loading dose was associated with higher peak neutrophil count and poor left ventricular systolic function, suggesting an important role of clopidogrel loading dose in the improvement of left ventricular function and high loading dose may exhibit better anti-inflammatory properties.

## 1. Introduction

Inflammation plays an important role in plaque development and left ventricular remodeling during acute myocardial infarction (AMI) [1, 2]. Several studies have shown an association between elevated levels of baseline neutrophils and poor heart function in patients with AMI [3, 4]. It has been recognized that platelet was a key contributor to initiate and propagate thrombosis and dual antiplatelet therapy with aspirin and clopidogrel has given significant benefits in patients with AMI [5, 6].

Evidence from clinical studies revealed that 600 mg loading dose of clopidogrel compared with 300 mg resulted in decreased 30-day ischemic adverse event and death rates in patients with ST-segment elevation myocardial infarction (STEMI) undergoing primary percutaneous coronary intervention (PPCI) [7, 8]. Numerous cross-links are known to exist between the thrombotic and inflammatory pathways in the pathophysiology of acute coronary syndrome (ACS) [1, 2]. As well, it has been proposed that clopidogrel may also exhibit some anti-inflammatory properties [9, 10]. However, there is lack of support for the association between high loading dose of clopidogrel and heart function in patients with PPCI. And until now, it is not clear whether the higher loading dose of clopidogrel has a better anti-inflammatory effect.

The aim of this study was to establish the association of loading dose of clopidogrel and left ventricular systolic function in patients with STEMI. Earlier study showed that neutrophil peaked within 24 hours after the onset of STEMI [11], so we wished to examine the association between peak neutrophil count and the benefit with high loading dose of clopidogrel.

## 2. Patients and Methods

### 2.1. Patients

Patients with STEMI were selected from the department of cardiology at the Peking University Third Hospital over the period from January 2008 to March 2011. Permission for the study was obtained by the local ethics committee. Written informed consent was obtained from the study population. STEMI was diagnosed according to the American College of Cardiology/American Heart Association guideline in 2004. All patients received successful PPCI (defined as coronary angiography with optimized flow of TIMI grade 3) within 12 h from symptom onset. Digital angiograms were analyzed by two independent, experienced interventional cardiologists. In order to assess coronary blood flow as a continuous variable, the corrected TIMI frame count (CTFC) was determined on final angiogram as described [12]. A total of 488 consecutive patients with STEMI were enrolled. The study excluded patients with (1) infectious disease (*n* = 44); (2) death or cardiogenic shock that happened during the hospitalization (*n* = 35); (3) usage of antiplatelet drugs prior to the onset (*n* = 27); (4) significant kidney or hepatic diseases (*n* = 17); (5) other causes of AMI (*n* = 6); (6) malignancy (*n* = 2). Thus, 357 patients (72.2% men, mean age 60 ± 11.3 years) constituted the present study.

### 2.2. Blood Sampling

Peripheral venous blood samples were obtained from all patients at the admission and in the morning of the first (D1), third (D3), and seventh day (D7) after STEMI (*n* = 357). The first blood sample was drawn prior to commencement of antiplatelet therapy. Blood samples were taken into standardized tubes (INSEPACK ST serials, Beijing, China) containing dipotassium ethylenediaminetetraacetate (EDTA-K2) and stored in room temperature. Total white blood cells (WBC) and neutrophils were measured 30 min after blood collection with an automated hematology analyzer (XE2100, Sysmex, Kobe, Japan). The reference ranges for total WBC and neutrophils are (4.0–10.0) × 10^9^/L and (3.0–5.0) × 10^9^/L, respectively. Blood samples for high-sensitive C-reactive protein (hs-CRP) and blood lipid analysis were taken between 24 and 48 h after admission. In a subgroup including all of the patients admitted during October 2009 to March 2011 (*n* = 52), additional blood samples were collected at the admission and 4–6 h, 12 h, 24 h, and the seventh day (D7) after PPCI. All samples were collected in vacuum blood collection tubes with clot activator and centrifuged at 3,000 rpm for 10 min at 4°C. An aliquot of the serum was stored at −80°C till analysis. Repeated freeze-thaw cycles were avoided.

### 2.3. Killip Classification

Killip class was evaluated by three clinicians during the first two days after STEMI according to the classic article [13]. Class 1: no evidence of heart failure; Class 2: signs indicating mild to moderate degree of heart failure (e.g., S3 gallop, rales < half-way up lung fields, or elevated jugular venous pressure); Class 3: pulmonary edema; and Class 4: cardiogenic shock or hypotension.

### 2.4. Assays

In a subgroup including all the patients admitted during October 2009 to March 2011 (*n* = 52), serum levels of interleukin-6 (IL-6) and interleukin-10 (IL-10) were measured by enzyme linked immunosorbent assay (ELISA) according to the manufacturer's instruction (ELISA kit, R&D Systems, Minneapolis, MN, USA). The minimal detection limits for IL-6 and IL-10 were 0.7 pg/mL and 3.9 pg/mL, respectively. These assays were performed by an investigator blinded to the sources of the samples.

### 2.5. Adjunctive Pharmacotherapy

All patients received 300 mg acetylsalicylic acid before intervention and unfractionated heparin during PPCI on routine basis. Different loading dose of clopidogrel (300 mg, 450 mg, or 600 mg) was given at the discretion of the clinician in the emergency room within 30 minutes after admission and subsequently 75 mg daily for the period of hospitalization. The high loading dose of clopidogrel is defined as 600 mg and the low loading dose is defined as 300 mg or 450 mg. The glycoprotein IIb/IIIa inhibitors (abciximab) were administered during PCI, at the discretion of the operator, as a 0.25 mg/kg bolus and a 0.125 ug/kg/min 12–24 h infusion. Other drugs commonly prescribed were angiotensin-converting enzyme inhibitors (ACEIs) or angiotensin II receptor blockers (ARBs), *β*-blockers, statins, and isonitrate.

### 2.6. Echocardiography

Each patient underwent echocardiography lying in the left decubitus position during the first three days after STEMI using a GE-VingMedVechocardiographic machine (Vivid 7) with a 3.3 MHz multiphase array probe. These examinations were performed by experienced cardiologists. The status of receiving *β*-blockers treatment, heart rates, and rhythm during the procedures were recorded. Left ventricular ejection fraction (LVEF) was obtained using a modified biplane version of Simpson's method with apical two- and four-chamber views. Low LVEF was defined as LVEF ≤ 50%. Regional wall motion abnormalities were evaluated by multiple apical and short-axis views as in routine clinical practice. The left ventricle was divided into 17 segments (6 basal, 6 midventricular, and 5 apical) as recommended by the American Society of Echocardiography [14]. Each segment was analyzed individually and scored by motion and systolic thickening as follows: 1 = normal/hyperkinesis, 2 = hypokinesis, 3 = akinesis, 4 = dyskinesis, and 5 = aneurysmal. Left ventricle wall motion score index (WMSI) was derived from the sum of all scores divided by the number of LV segments [14].

### 2.7. Statistics

Baseline clinical parameters between the group of low loading dose of clopidogrel and the group of high loading dose were compared using Student's unpaired* t*-tests, chi-squared tests, or Mann-Whitney test as appropriate. To test the normal distribution, the Kolmogorov-Smirnov test was used. One-way analysis of variance followed by post hoc analysis was used for comparing total WBC and neutrophils among all patients with STEMI at the admission, D1, D3, and D7. With 44 patients in both groups, we can detect the 0.07 difference of LVEF between two groups with 90% power [15] (the mean of LVEF in the Peking University Third Hospital from January 2005 to January 2008 is 54.1%, and the SD of LVEF is 8.4%). And because this study is nonrandomized, the minimum of each group is doubled. A logistic regression model was used to assess the association between clopidogrel loading dose and LVEF, adjusting for confounders (age, sex, diabetes mellitus, hypertension, anterior myocardial infarction, time from onset to admission, preinfarction angina, corrected TIMI frame count, fasting blood glucose levels, hs-CRP protein, and clopidogrel loading dose). Backward selection procedure with* P* level more than 0.1 for exclusion from the model and* P* level less than 0.05 for staying in the model was used to select important predictors. The final models were evaluated by using odds ratio (OR) and 95% confidence interval (CI). Statistical significance was defined as *P* < 0.05. All analyses were performed using SPSS version 15.0 (SPSS, Chicago, IL).

## 3. Results

### 3.1. Time Course of WBC and Neutrophil (*n* = 357)

**Figure 1:**
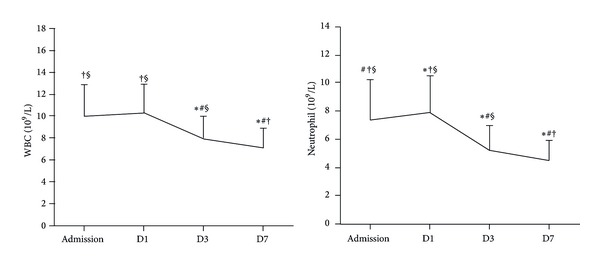
The time course of WBC and neutrophil in patients with STEMI who received successful PPCI (*n* = 357). ∗ Represents *P* < 0.05 compared with admission, # represents *P* < 0.05 compared with D1, † represents *P* < 0.05 compared with D3, and § represents *P* < 0.05 compared with D7. Note: D1: the first day, D3: the third day, and D7: the seventh day.

Figure 1 shows the time course of WBC and neutrophil from admission to D7 after AMI. Total WBC counts on D1 (10.33 ± 2.63 × 10^9^/L) were higher than that at the admission (9.97 ± 2.91 × 10^9^/L), but there was no significance. Neutrophils reached their peak values on D1 (7.92 ± 2.60 × 10^9^/L) and then reduced from D3 to D7 (*F* = 145.851, *P* < 0.0001).

### 3.2. Baseline Characteristics (*n* = 357)

Three hundred and fifty-seven patients enrolled in the study. The ratio of receiving low clopidogrel loading dose to high loading dose was 2.5 : 1, 2.4 : 1, 3.2 : 1, and 1.8 : 1 in 2008, 2009, 2010, and 2011, respectively. The clinical characteristics and laboratory findings of the patients of different clopidogrel loading dose are summarized in Table 1. PPCI was successfully performed in all patients. The mean interval from the onset of AMI to arrival at the hospital was 4.8 ± 3.9 h. Risk factor profiles and some baseline clinical parameters such as male percentage, BMI, blood pressure, lipid profile, and concomitant illness were comparable between the two groups. The low clopidogrel loading dose group had higher hs-CRP than the group of high clopidogrel loading dose (Table 1).

### 3.3. IL-6 and IL-10 Levels in a Subgroup (*n* = 52)

In a subgroup including the patients with AMI who were admitted during October 2009 to March 2011 (*n* = 52), the levels of IL-6 obtained 4–6 h after PPCI were significantly higher in the low clopidogrel loading dose group than those found in the group of high clopidogrel loading dose, while the IL-10 levels showed inverse changes (shown in Table 2). On other time points, the levels of IL-6 and IL-10 were almost comparable between the two groups (Table 2). Baseline clinical parameters were comparable between the two groups (Table 2).

### 3.4. WBC, Neutrophil, and Platelet Related Indices (*n* = 357)

**Figure 2:**
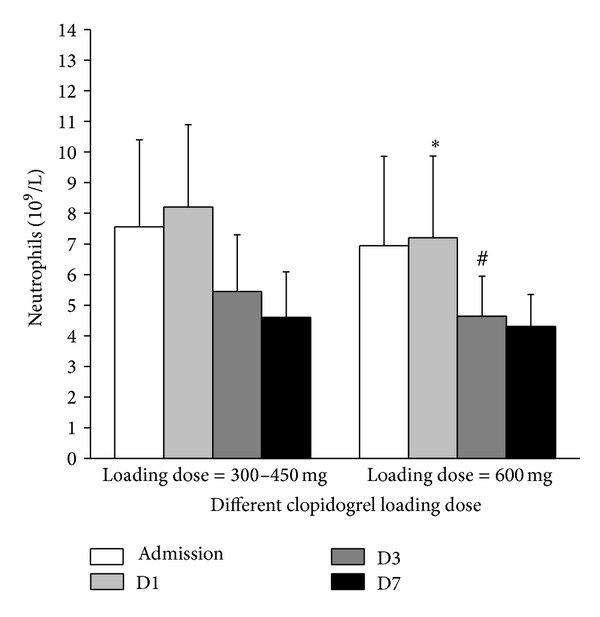
Comparison of neutrophil between different clopidogrel loading doses (low clopidogrel loading dose: 300–450 mg, *n* = 259; high clopidogrel loading dose: 600 mg, *n* = 98) at different time points during the first week (*n* = 357). ∗ Represents *P* < 0.05 compared with low clopidogrel loading dose (300–450 mg) on D1 and # represents *P* < 0.05 compared with low clopidogrel loading dose (300–450 mg) on D3.

The higher levels of WBC and neutrophil on D1 and D3 were found in patients of low clopidogrel loading dose as shown in Table 3 and Figure 2. Platelet related indices were comparable between the two groups (Table 3).

### 3.5. Heart Function Related Indices (*n* = 357)

Heart function related indices of different clopidogrel loading dose were shown in Table 4. Compared with high clopidogrel loading dose group, patients with low loading dose were more likely to suffer from Killip class >1. The low clopidogrel loading dose group had lower FS and LVEF than the group of high loading dose. Moreover, patients of low loading dose had higher Nt-proBNP levels, LVESd, and WMSI than the patients of high loading dose. There was no significant difference in LVEDd, CK, and CK-MB levels between the two groups. Receiving *β*-blockers treatment, heart rates, and atrial fibrillation occurrence during the procedures were comparable between the two groups. A logistic regression model was used to assess the association between clopidogrel loading dose and LVEF, adjusting for confounders (age, sex, diabetes mellitus, hypertension, anterior myocardial infarction, time from onset to admission, preinfarction angina, corrected TIMI frame count, fasting blood glucose levels, hs-CRP, and clopidogrel loading dose). After adjusting for the above factors, low clopidogrel loading dose remained an independent predictor of low LVEF (LVEF ≤ 50%) as data show in Table 5.

## 4. Discussion

The present study found that, in patients with STEMI undergoing successful PPCI, higher levels of peak neutrophil and lower LVEF were found in patients of low clopidogrel loading dose group (300 mg or 450 mg). After adjusting for the confounders, low clopidogrel loading dose remained an independent predictor of low LVEF (LVEF ≤ 50%). Also, the current study observed that total WBC and neutrophil count changed dramatically during the first week: neutrophil reached their peak values on the first day after STEMI and then reduced from the third day to the seventh day; total WBC showed the similar trend.

AMI is universally accompanied by a transient elevation of WBC as part of the inflammatory response [16, 17]. Inflammation has been shown to mediate healing and scar formation after termination of the ischemic event as well as progressive development of tissue damage and ventricular remodeling [18–21]. The first leukocytes which infiltrate the infracted myocardium are neutrophils [19]. Moreover, neutrophils are widely recognized as important mediators of myocardial and vascular injury [19, 22, 23]. Evidence from clinical studies revealed that elevation of baseline neutrophil count is associated with higher in-hospital and short-term mortality in patients with AMI [24, 25]. In keeping with previous studies, total WBC and neutrophil count in the present study reached their peak values on the first day after AMI. Based on what was discussed earlier, peak leukocyte and neutrophil count might better reflect the magnitude of the inflammatory response to myocardial necrosis [16]. PPCI were performed in all patients in this study. Due to the fact that the “no-reflow” phenomenon has also been directly linked to neutrophil localization, the neutrophil peak may be related to the reperfusion injury [26].

In the present study higher levels of peak neutrophil and lower LVEF were found in patients of low clopidogrel loading dose group. As has been demonstrated in clinical experiments, increased neutrophil at the admission in patients with AMI was related to the early development of congestive heart failure [3, 24]. There is abundant evidence that leukocytes contribute to impairment of myocardial function and adverse ventricular remodeling in the postinfarction setting [20, 21]. Neutrophils are known to accumulate in the ischemic and reperfused areas and might release proteolytic enzymes or reactive oxygen species to injure surrounding myocytes [19]. Moreover, the “no-reflow” phenomenon could be interpreted as a consequence of neutrophil and platelet localization [26]. It is well known that the no-reflow phenomenon is associated with poor left ventricular function [27, 28].

Platelet activation plays a crucial role in initiating and propagating thrombosis, and antiplatelet therapy is the key management of AMI [5, 6]. Experimental studies have demonstrated that there are increased interactions between platelets and leukocytes in the pathophysiology of AMI [1, 2]. Both leukocytes and platelets adhere to the endothelial lining at the culprit site of coronary artery occlusion [29]. In response to ischemia and reperfusion, neutrophils migrate into the vessel wall and microvasculature and secrete a variety of autacoids, which lead to vasoconstriction and platelet aggregation, such as thromboxane-B2 and leukotrienes-B4 [19]. Interactions between leukocytes and platelets amplify through upregulation of the CD11b/18 receptor on the leukocyte surface, inducing further release of cytokines and procoagulants [30]. In addition, platelet-neutrophils aggregates may also contribute to microvascular plugging and microembolization and mediate the “no-reflow” phenomenon [26].

Clopidogrel, an ADP receptor antagonist, is established to reduce the incidence of stroke, myocardial ischemia, or vascular death [5, 6, 31]. Several clinical trials have demonstrated that a loading dose of 600 mg clopidogrel significantly reduces the risk of major cardiovascular events (including death, ACS, revascularization, and stroke) without increase in major bleeding compared to 300 mg in patients undergoing PPCI [7, 8]. Since clopidogrel has inhibitory effect on platelets in a dose-dependent manner, dosing of clopidogrel is an important variable influencing the patient's prognosis [32]. Rapid and sufficient platelet inhibition is achieved 2 h after a 600 mg loading dose of clopidogrel [33].

A large scale study including 56,944 patients showed that clopidogrel was associated with reduced mortality in patients with heart failure who do not undergo PCI after their first-time AMI, whereas this association was not apparent in patients without heart failure [34]. Compared with application of aspirin and continuous infusion of unfractionated heparin, the intensified clopidogrel loading and maintenance doses provided early reperfusion and suppression of inflammatory response in patients with standard fibrinolysis therapy [24]. Specifically, an antiplatelets therapy, such as clopidogrel, has been proven to reduce circulating platelet-leukocyte conjugates [10].

It has been shown that platelet size, measured as mean platelet volume (MPV), is related to its reactivity [35]. In the present study, MPV was comparable between the low and high clopidogrel loading dose groups (*n* = 357). There was no difference in the IL-6 and IL-10 levels between the low and high clopidogrel loading dose groups on admission in a subgroup (*n* = 52), whereas 4–6 h after PPCI the levels of IL-6 were higher in the low clopidogrel loading dose group than those found in the group of high loading dose, the same time at which the IL-10 levels showed inverse changes. IL-6 and IL-10 have been established as important pro- and anti-inflammatory cytokines during acute myocardial ischemia [36, 37]. As discussed in the previous section, antiplatelets therapies, such as clopidogrel, may also exhibit some anti-inflammatory properties, thereby potentially offering benefits beyond inhibition of platelet activation [38].

Clopidogrel application before primary PCI (in the emergency department or the intensive care unit) was independently associated with a higher TIMI myocardial perfusion rate [39]. Based on the multiple effects of clopidogrel and its dose-dependent manner, it is easy to understand that high loading dosage of clopidogrel was associated with lower peak neutrophil and better left ventricular systolic function. Until then, on the basis of our observational data, as well as those from previous studies of patients with STEMI, it would seem prudent to treat patients with STEMI with high-dose clopidogrel.

The major limitation of the present study was its nonrandomized nature; the clopidogrel loading dose was left to the discretion of the treating physician. Thus, although the treatment effects reflect real-life practice, they might also reflect physician bias. In an attempt to control for potential confounders, we performed a multivariate analysis, the results of which support the unadjusted results. As the current study excluded the patients who did not perform primary PCI, the results of the present study only reflect the special population. Last, because of small sample size, the analysis of subgroup and in-hospital cardiovascular events could not be performed.

## 5. Conclusion

Higher peak neutrophil count and poor left ventricular systolic function were found in low clopidogrel loading dose. Furthermore, low loading dose of clopidogrel remained a significant predictor of low LVEF after adjusting for potential confounders. These results suggest an important role of clopidogrel loading dose in the improvement of left ventricular function and high loading dose may exhibit better anti-inflammatory properties.

## Figures and Tables

**Table 1:** Baseline characteristics.

Variable	Low clopidogrel loading dose (300–450 mg) (*n* = 259)	High clopidogrel loading dose (600 mg) (*n* = 98)	*P* value
Age (yrs)	61.4 ± 13.3	61.1 ± 12.7	0.84
Male sex (%)	79.9	82.7	0.65
Hypertension (%)	52.1	51.0	0.91
Diabetes mellitus (%)	35.9	28.6	0.21
Hypercholesterolemia (%)	37.5	33.7	0.54
Current smoking (%)	48.3	55.1	0.29
Family history of CAD (%)	14.7	15.3	0.87
Body mass index (kg/m^2^)	26.1 ± 4.2	25.5 ± 3.8	0.45
Anterior infarction (%)	51.9	41.8	0.10
Prior angina (%)	65.3	55.1	0.07
Systolic blood pressure (mm Hg)	134.3 ± 25.9	138.8 ± 31.3	0.23
Diastolic blood pressure (mm Hg)	76.8 ± 16.6	80.9 ± 19.7	0.13
Time to admission (h)	4.8 ± 3.7	4.9 ± 4.5	0.95
ST-segment resolution ≥50% within 120 minutes after PPCI (%)	87.6	87.6	1.00
Corrected TIMI frame count	32.1 ± 19.0	30.0 ± 18.5	0.36
Hemoglobin (g/L)	144.3 ± 19.1	145.6 ± 16.4	0.58
hs-CRP (mg/L)∗	**9.11 (3.23, 23.35)**	**4.20 (1.79, 17.97)**	0.03
Creatinine (*μ*mol/L)	86.7 ± 21.0	87.3 ± 21.5	0.83
Serum glucose (mmol/L)	6.74 ± 3.01	6.20 ± 2.37	0.13
Total cholesterol (mmol/L)	4.69 ± 1.03	4.63 ± 0.83	0.58
Triglyceride (mmol/L)	1.73 ± 0.92	1.91 ± 1.19	0.15
HDL-C (mmol/L)	0.95 ± 0.24	0.90 ± 0.20	0.06
LDL-C (mmol/L)	3.02 ± 0.90	2.92 ± 0.73	0.33
IIb/IIIa inhibitors	32.0	28.6	0.70

Values represent mean ± SD or the percent of the patients. *Values represent median with 25 and 75 percentiles.

Note: CAD: coronary artery disease; PPCI: primary percutaneous coronary intervention; hs-CRP: high-sensitive C-reactive protein; HDL-C: high density lipoprotein cholesterol; LDL-C: low density lipoprotein cholesterol.

**Table 2:** Baseline characteristics and IL-6 and IL-10 levels in a subgroup.

Variable	Low clopidogrel loading dose (300–450 mg) (*n* = 32)	High clopidogrel loading dose (600 mg) (*n* = 20)	*P* value
Age (yrs)	58.4 ± 15.3	65.7 ± 12.2	0.17
Male sex (%)	84.3	55.0	0.20
Hypertension (%)	37.5	40.0	0.53
Diabetes mellitus (%)	21.9	20.0	1.00
Hypercholesterolemia (%)	31.3	25.0	1.00
Current smoking (%)	50.0	35.0	0.76
Body mass index (kg/m^2^)	26.8 ± 4.7	24.7 ± 4.8	0.17
Anterior infarction (%)	40.6	30.0	0.34
IL-6 (ng/L)			
Admission	7.51 (5.13, 12.93)	5.86 (4.55, 8.08)	0.20
4–6 h after PPCI	10.31 (7.90, 14.89)	5.96 (5.18, 10.93)	0.02
12 h after PPCI	8.43 (6.88, 11.90)	8.52 (6.81, 13.84)	0.88
24 h after PPCI	7.79 (6.16, 11.15)	6.70 (5.72, 8.56)	0.24
7 d after PPCI	9.73 (6.40, 13.53)	8.13 (6.56, 12.11)	0.73
Peak value	13.44 (11.94, 19.90)	11.54 (8.94, 24.59)	0.24
IL-10 (ng/L)			
Admission	18.53 (15.54, 22.73)	21.78 (20.74, 23.16)	0.11
4–6 h after PPCI	16.39 (11.24, 19.56)	20.64 (16.87, 22.57)	0.02
12 h after PPCI	18.73 (15.70, 20.99)	19.06 (15.36, 21.52)	0.86
24 h after PPCI	18.29 (15.05, 21.22)	20.72 (18.87, 22.55)	0.05
7 d after PPCI	17.66 (15.59, 20.86)	19.44 (16.77, 21.15)	0.50
Peak value	21.35 (20.00, 24.33)	22.71 (21.58, 24.60)	0.12

Values represent mean ± SD or median with 25 and 75 percentiles.

Note: CAD: coronary artery disease; PCI: percutaneous coronary intervention; hs-CRP: high-sensitive C-reactive protein; HDL-C: high density lipoprotein cholesterol; LDL-C: low density lipoprotein cholesterol; IL-6: interleukin-6; IL-10: interleukin-10.

**Table 3:** WBC, neutrophil, and platelet related indices.

Variable	Low clopidogrel loading dose (300–450 mg) (*n* = 259)	High clopidogrel loading dose (600 mg) (*n* = 98)	*P* value
Total WBC (×10^9^/L)			
Admission	10.14 ± 2.87	9.47 ± 2.99	0.07
D1	10.58 ± 2.66	9.70 ± 2.43	0.01
D3	8.15 ± 2.11	7.42 ± 1.76	0.01
D7	7.23 ± 1.84	6.94 ± 1.52	0.25
Neutrophil (×10^9^/L)			
Admission	7.56 ± 2.84	6.94 ± 2.92	0.09
D1	8.21 ± 2.68	7.20 ± 2.67	0.001
D3	5.45 ± 1.85	4.64 ± 1.31	<0.0001
D7	4.60 ± 1.49	4.30 ± 1.05	0.08
Platelet counts (×10^9^/L)			
Admission	225.5 ± 60.0	208.2 ± 56.3	0.02
D1	209.2 ± 54.5	197.7 ± 57.2	0.08
D3	191.0 ± 50.3	178.4 ± 56.6	0.08
D7	222.9 ± 54.2	214.5 ± 55.5	0.29
MPV (fL)			
Admission	10.16 ± 0.99	10.05 ± 1.12	0.39
D1	8.31 ± 0.89	8.48 ± 0.89	0.10
D3	8.53 ± 0.94	8.56 ± 0.90	0.82
D7	8.38 ± 0.87	8.53 ± 0.95	0.25

Values represent mean ± SD.

Note: WBC: white blood cell; D1: the first day after AMI; D3: the third day; D7: the seventh day; MPV: mean platelet volume.

**Table 4:** Heart function related indices.

Variable	Low clopidogrel loading dose (300–450 mg) (*n* = 259)	High clopidogrel loading dose (600 mg) (*n* = 98)	*P* value
Peak CK (IU/L)	2350.4 ± 1933.7	2046.9 ± 1766.0	0.18
Peak CK-MB (IU/L)	228.2 ± 147.5	210.7 ± 148.9	0.32
Nt-proBNP (pg/mL)	2166.8 ± 2922.0	1112.8 ± 1176.7	<0.0001
Killip class >1 (%)	25.1	12.2	0.01
Echocardiographic findings			
Heart rates (beat/min)	75.6 ± 11.3	74.7 ± 9.6	0.28
Atrial fibrillation (%)	21.0	18.6	0.66
On *β*-blocker treatment (%)	73.1	70.0	0.33
LVEDd (mm)	49.8 ± 5.1	49.1 ± 4.8	0.26
LVESd (mm)	35.0 ± 5.8	33.4 ± 5.2	0.02
FS (%)	29.9 ± 7.1	31.9 ± 7.5	0.02
LVEF (%)	53.2 ± 8.4	56.4 ± 7.9	0.002
WMSI	1.56 ± 0.21	1.47 ± 0.23	0.001

Values represent mean ± SD or the percent of the patients.

Note: CK: creatine kinase; CK-MB: creatine kinase-MB; Nt-proBNP: N-terminal pro B-type natriuretic peptide; LVEDd: left ventricular end diastolic dimension; LVESd: left ventricular end systolic dimension; FS: fractional shortening; LVEF: left ventricular ejection fraction; WMSI: wall motion score index.

**Table 5:** Multiple logistic analysis of low LVEF (LVEF ≤50%).

Variable	OR	95% CI	*P* value
Anterior infarction	5.15	2.89–9.20	<0.0001
Diabetes mellitus	2.48	1.40–4.41	0.002
Time to admission	0.92	0.86–0.98	0.02
Low clopidogrel loading dose (300–450 mg)	1.97	1.03–3.79	0.04
